# Supplementary material for: Antibiotic resistant bacteria survive treatment by doubling while shrinking
Source: mBio. 2024 Nov 20;15(12):e02375-24. doi: 10.1128/mbio.02375-24 (PMC11633386; doi:10.1128/mbio.02375-24)
Supplement: Supplemental material — Supplemental figures and captions for Table S1 and Videos S1 to S3. [file mbio.02375-24-s0001.docx]

**Supplemental Material for**

**Antibiotic resistant bacteria survive treatment by doubling while shrinking**

Adrian Campey,^1^ Urszula Łapińska,^1^ Remy Chait,^1^ Krasimira Tsaneva-Atanasova,^1,2^ Stefano Pagliara^1,*^

^1^Living Systems Institute and Biosciences, University of Exeter, Exeter, Devon, EX4 4QD, United Kingdom

^2^EPSRC Hub for Quantitative Modelling in Healthcare, University of Exeter, Exeter, EX4 4QJ, UK

*Correspondence: [s.pagliara@exeter.ac.uk](mailto:s.pagliara@exeter.ac.uk)

**Figure S1. Impact of the exposure to sub-MIC ciprofloxacin concentrations on bacterial growth in the structured environment.** Temporal dependence of *E. coli* expansion in the structured environment in the presence of different concentrations of ciprofloxacin: no drug (black squares), 10% (red upwards triangles), 25% (blue circles), 50% (orange downwards triangles), or 100% (purple diamonds) the MIC of ciprofloxacin against the parental strain *E. coli* BW25113. Bacterial expansion was measured as the increasing distance from the inoculation site at t = 0. Symbols and error bars are the mean and standard error obtained by averaging measurements performed in biological triplicate.


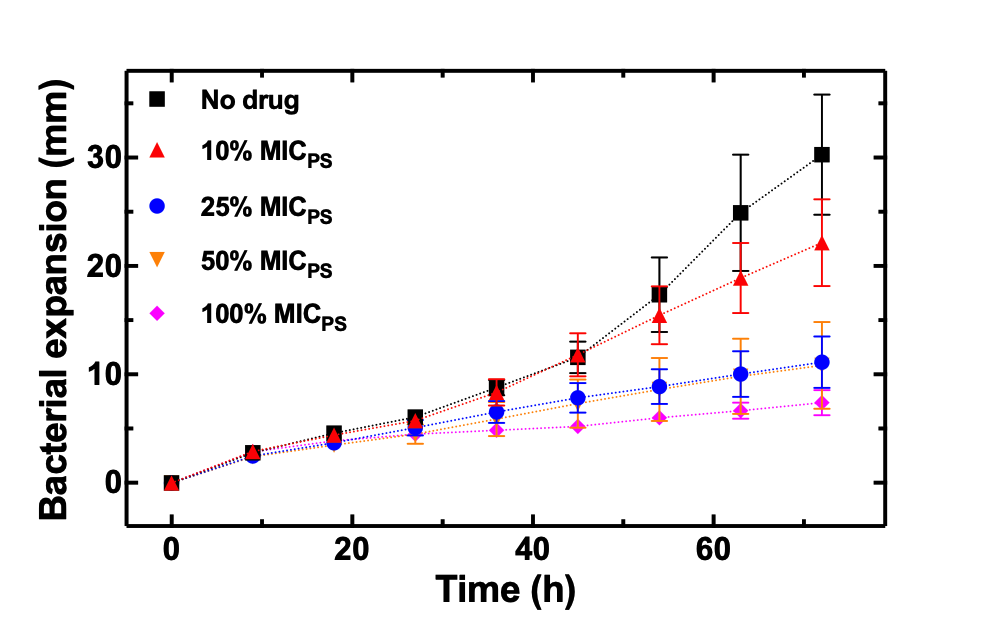


**Figure S2. Impact of the exposure to sub-MIC ciprofloxacin concentrations on bacterial growth in the well-mixed environment.** Temporal dependence of *E. coli* growth in the well-mixed environment in the presence of different concentrations of ciprofloxacin: no drug (black squares), 10% (red upwards triangles), 25% (blue circles), 50% (orange downwards triangles), 100% (purple diamonds) the MIC of ciprofloxacin against the parental strain *E. coli* BW25113. Bacterial growth was quantified by measuring optical density (O.D.) at 600 nm on aliquots taken from the growing cultures at each time point. Symbols and error bars are the mean and standard error obtained by averaging measurements performed in biological triplicate. Cultures were diluted 1:100 into 100 mL of LB medium containing the appropriate ciprofloxacin concentration every 24 h and this is reflected in the decrease in O.D. at t = 24 h and 48 h.


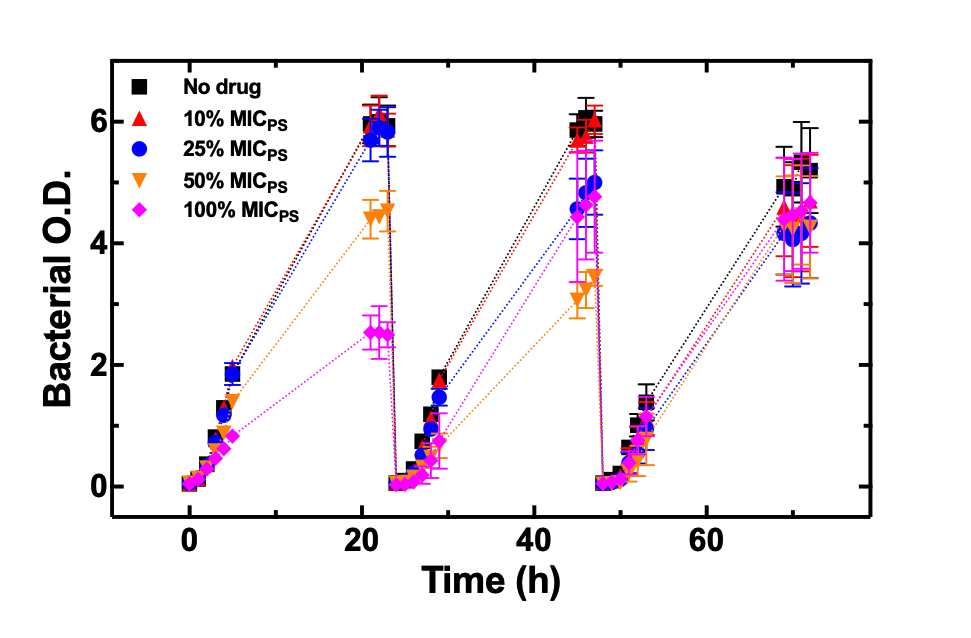

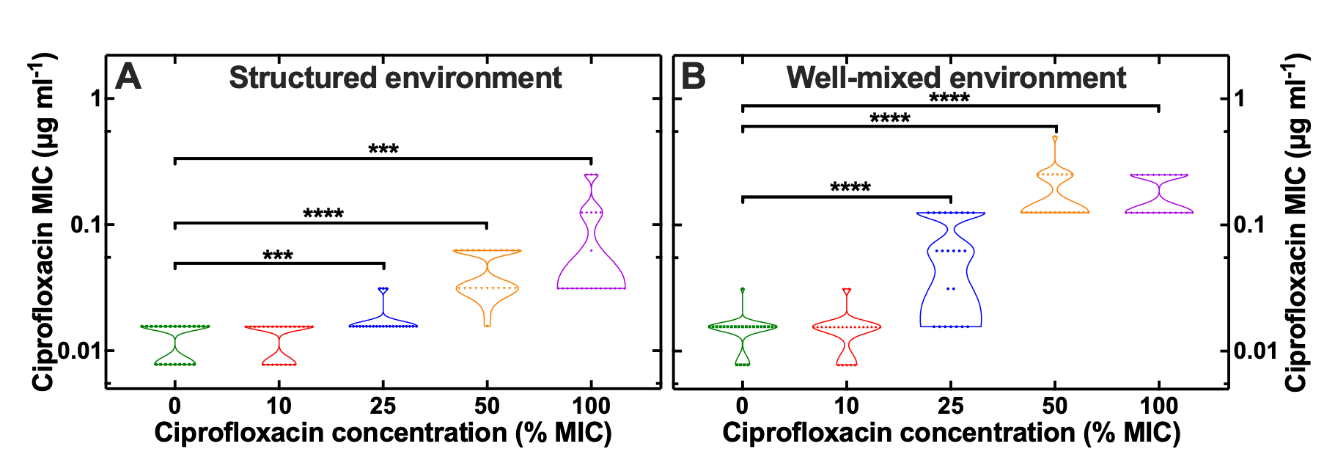


**Figure S3. Impact of the environmental structure on the emergence of genetic resistance to ciprofloxacin.** Dependence of the emergence of resistance to ciprofloxacin on the concentration of ciprofloxacin experienced by *E. coli* during evolutionary experiments in (a) the structured and (b) the well-mixed environment. Each symbol represents the ciprofloxacin MIC value measured for one out of eight technical replicates from three different evolutionary experiments for a total of 24 measured ciprofloxacin MIC values for each environmental condition. ***: p <0.001; ****: p <0.0001.

**Figure S4. Impact of the duration of ciprofloxacin selection pressure on the maintenance of resistance to ciprofloxacin.** Dependence of the capability of resistant mutants to maintain resistance to ciprofloxacin on the duration of ciprofloxacin selection pressure at the end of evolutionary experiments in the structured (blue filled circles) or well-mixed environment (open red circles) using ciprofloxacin at (a) 25%, (b) 50% or (c) 100% its MIC against the *E. coli* parental strain (MIC_PS_). At the end of each evolutionary experiment, we either kept using ciprofloxacin (continuous) or removed ciprofloxacin from the environment either for 2 h (i.e. when the mutant culture was growing to exponential phase before the microbroth serial dilution assay), for 17 h (i.e. when the mutant culture was growing overnight for biomass expansion), or for 19 h (both during overnight culture and following exponential growth). Each symbol represents the ciprofloxacin MIC value measured in eight technical replicates for each mutant indicated in figure. **: p <0.01; ***: p <0.001; ****: p <0.0001.


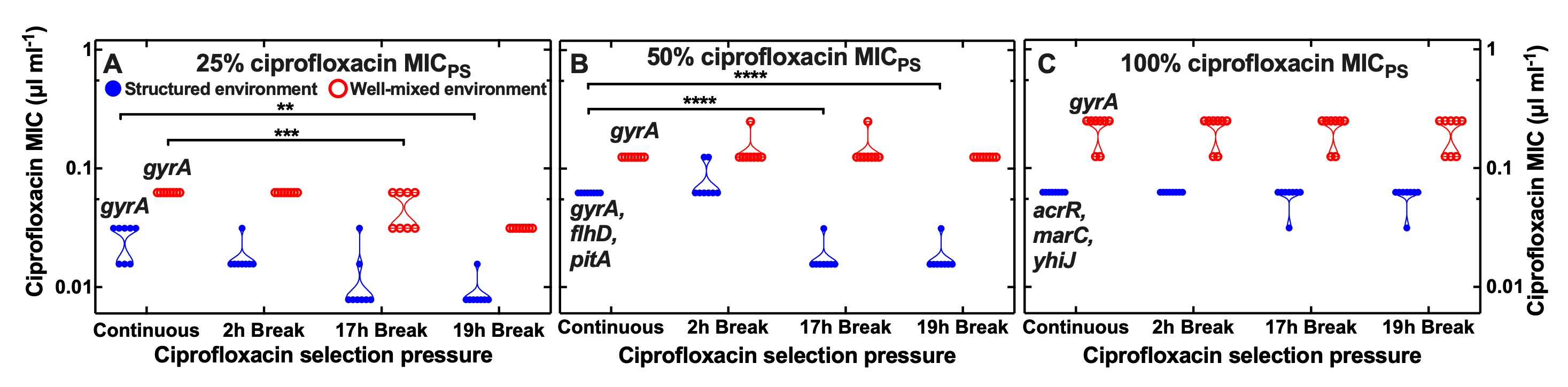


**Figure S5. Impact of the structure of the environment on tolerance to ciprofloxacin in *E. coli* evolved in the absence of ciprofloxacin.** Temporal dependence of bacterial counts for *E. coli* obtained from evolutionary experiments in the absence of ciprofloxacin in (a) the structured and (b) the well-mixed environment after exposure to 1× (blue circles), 10× (red squares), or 25× (green triangles) the MIC_PS_ value. The log survival at each time point was calculated as the mean and standard deviation of the ratio of the colony forming units at that time point divided by the corresponding colony forming units at t = 0 performed in biological triplicate.


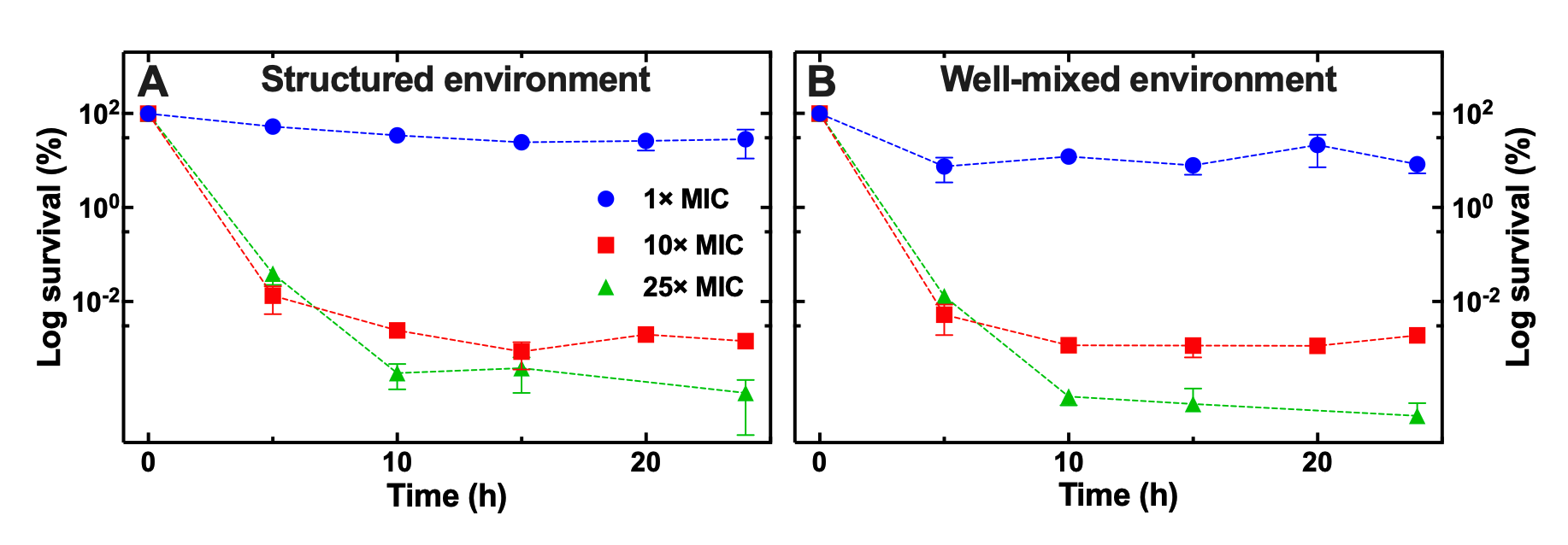


**Table S1. Further mutations occurring in each evolutionary experiment.** Structure of the environment and ciprofloxacin concentration as a percentage of the MIC_PS_ value employed during each triplicate evolutionary experiments. Corresponding MIC fold change of each mutant compared with the parental strain, mutation position, type, frequency, annotation, gene and description of gene product.

**Video S1** Time-lapse imaging of the parental strain *E. coli* BW25113 growing on agar plates in the absence of ciprofloxacin or in the presence of ciprofloxacin at a concentration of 10%, 25%, 50% or 100% its MIC against *E. coli* BW25113. Each plate was inoculated with a single colony of *E. coli* BW25113 at two sites, using the numbered grid background to ensure consistency of inoculation site across the plates.

**Video S2** Time-lapse microscopy images of individual cells of the 4-fold resistant triple mutant from the structured environment (with mutations in the genes *acrR*, *marR* and *yhiJ*) exposed to LB medium (0 < t < 120 min), ciprofloxacin at 25× the MIC_PS_ value (120 min < t < 360 min) and LB medium (360 min < t < 450 min) within the microfluidic mother machine.

**Video S3** Time-lapse microscopy images of individual cells of the 16-fold resistant *gyrA* S83L mutant from the well-mixed environment exposed to LB medium (0 < t < 120 min), ciprofloxacin at 25× the MIC_PS_ value (120 min < t < 360 min) and LB medium (360 min < t < 450 min) within the microfluidic mother machine.
