# Supplementary figures and images for: Antibiotic resistant bacteria survive treatment by doubling while shrinking
Source: mBio. 2024 Nov 20;15(12):e02375-24. doi: 10.1128/mbio.02375-24 (PMC11633386; doi:10.1128/mbio.02375-24)

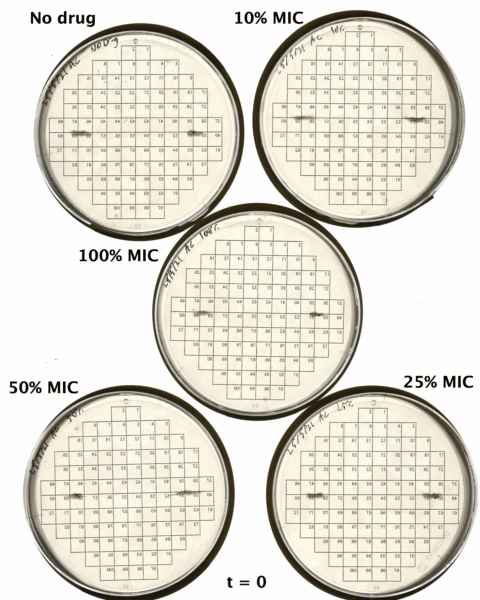

Supplement: Video S1 — Time-lapse imaging of the parental strain E. coli BW25113 growing on agar plates in the absence or presence of ciprofloxacin. [file mbio.02375-24-s0003.gif]

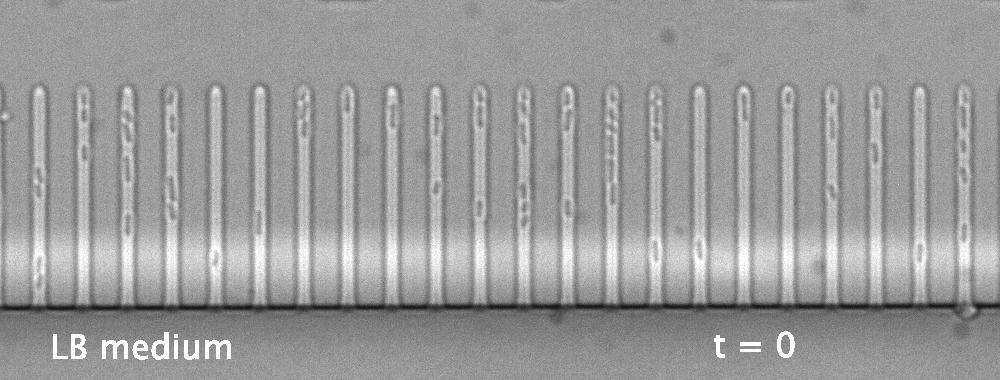

Supplement: Video S2 — Time-lapse microscopy images of individual cells of the 4-fold resistant triple mutant from the structured environment. [file mbio.02375-24-s0004.gif]

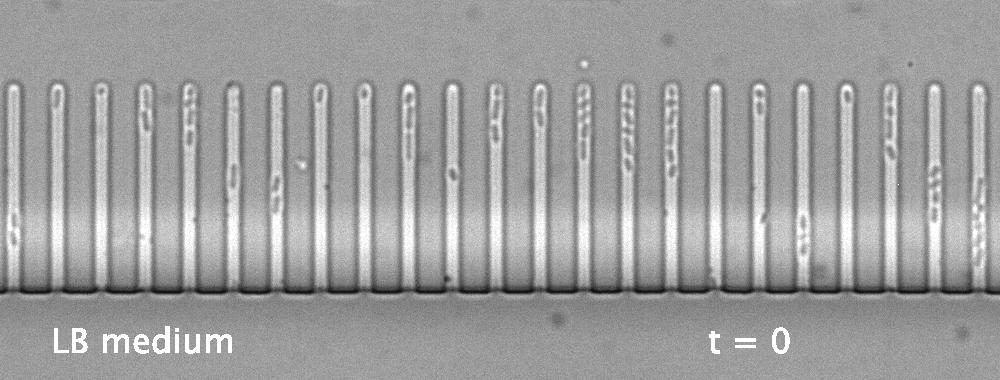

Supplement: Video S3 — Time-lapse microscopy images of individual cells of the 16-fold resistant gyrA S83L mutant from the well-mixed environment. [file mbio.02375-24-s0005.gif]
